# Warmth suppresses and desensitizes damage-sensing ion channel TRPA1

**DOI:** 10.1186/1744-8069-8-22

**Published:** 2012-03-29

**Authors:** Sen Wang, Jongseok Lee, Jin Y Ro, Man-Kyo Chung

**Affiliations:** 1 Department of Neural and Pain Sciences, School of Dentistry, Program in Neuroscience, University of Maryland, 650 W. Baltimore St., Baltimore, MD 21201, USA

**Keywords:** TRPA1, Pain, Temperature

## Abstract

**Background:**

Acute or chronic tissue damage induces an inflammatory response accompanied by pain and alterations in local tissue temperature. Recent studies revealed that the transient receptor potential A1 (TRPA1) channel is activated by a wide variety of substances that are released following tissue damage to evoke nociception and neurogenic inflammation. Although the effects of a noxious range of cold temperatures on TRPA1 have been rigorously studied, it is not known how agonist-induced activation of TRPA1 is regulated by temperature over an innocuous range centred on the normal skin surface temperature. This study investigated the effect of temperature on agonist-induced currents in human embryonic kidney (HEK) 293 cells transfected with rat or human TRPA1 and in rat sensory neurons.

**Results:**

Agonist-induced TRPA1 currents in HEK293 cells were strongly suppressed by warm temperatures, and almost abolished at 39°C. Such inhibition occurred when TRPA1 was activated by either electrophilic or non-electrophilic agonists. Warming not only decreased the apparent affinity of TRPA1 for mustard oil (MO), but also greatly enhanced the desensitization and tachyphylaxis of TRPA1. Warming also attenuated MO-induced ionic currents in sensory neurons. These results suggest that the extent of agonist-induced activity of TRPA1 may depend on surrounding tissue temperature, and local hyperthermia during acute inflammation could be an endogenous negative regulatory mechanism to attenuate persistent pain at the site of injury.

**Conclusion:**

These results indicate that warmth suppresses and desensitizes damage-sensing ion channel TRPA1. Such warmth-induced suppression of TRPA1 may also explain, at least in part, the mechanistic basis of heat therapy that has been widely used as a supplemental anti-nociceptive approach.

## Background

Transient receptor potential A1 (TRPA1) is a Ca^2+^-permeable non-selective cationic channel enriched in a subpopulation of nociceptive sensory neurons [1,2]. The activation of TRPA1 directly evokes pain and induces vasodilation and neurogenic inflammation. TRPA1 can be activated by a wide range of irritants including mustard oil (MO), cinnamaldehyde, and formaldehyde. Endogenous products generated by tissue damage and oxidative stress, such as H_2_O_2_, 4-hydroxynonenal, prostaglandin J_2_, and reactive oxygen and nitrogen species can also activate TRPA1 [3]. Thus, TRPA1 functions as a sensor of endogenous tissue damage and exogenous harmful compounds, and is implicated in multiple pathological conditions, including chronic pain and respiratory and cardiovascular diseases [3-5]. Recently, a gain-of-function mutation of TRPA1 N855S was found to cause familial episodic pain syndrome [6], further suggesting a role for TRPA1 in nociception.

Many agonists activate TRPA1 by covalent binding to reactive residues located at an intracellular amino terminal domain [7,8]. Since the covalent modification of TRPA1 by an electrophilic agonist is not readily reversible after washout, but persists for more than an hour [8], TRPA1 activation by reactive agonists typically show prolonged residual activity even following washout [7-9]. Thus TRPA1 may be persistently activated by endogenous agonists that are released at the site of injury or inflammation [10-14]. Consistent with this notion, specific antagonists against TRPA1 reverse persistent pain under various pathological conditions in experimental animals [15-18]. Therefore, developing a more effective approach to suppress the activity of TRPA1 may be beneficial in the treatment of chronic pain conditions.

Since mammalian TRPA1 was originally proposed as a noxious cold sensor [2], the activation of TRPA1 has been rigorously studied over a cold temperature range. Although cold sensitivity of TRPA1 is controversial [19], a recent study reported a potentiating effect of cold on agonist-induced activation of TRPA1, suggesting it has a role in cold hyperalgesia rather than cold pain [20]. However, it is not known how the agonist-induced activation of TRPA1 is affected by temperature changes at the skin surface. Moreover, acute or chronic inflammatory conditions in human and experimental animals are accompanied by alterations in the local tissue temperature [21-24]. Therefore, investigating the effects of temperature at the skin surface on agonist-induced activation of TRPA1 should provide information about TRPA1 activity under conditions that are more pathophysiologically relevant.

In this study, we assessed the effect of the temperature on agonist-induced activation of TRPA1 in vitro.

## Results

### Agonist activation of TRPA1 is strongly suppressed by warmth

To investigate the modulation of agonist-induced activation of TRPA1 over physiologically relevant temperature range, we analysed MO-evoked currents at various temperatures using the whole-cell voltage clamp technique. In order to better quantify the activity of recombinant TRPA1, we generated human embryonic kidney (HEK) 293 cell lines stably expressing either rat or human TRPA1. Unless otherwise indicated, data presented in the Results were obtained from cells stably expressing TRPA1.

**Figure 1:**
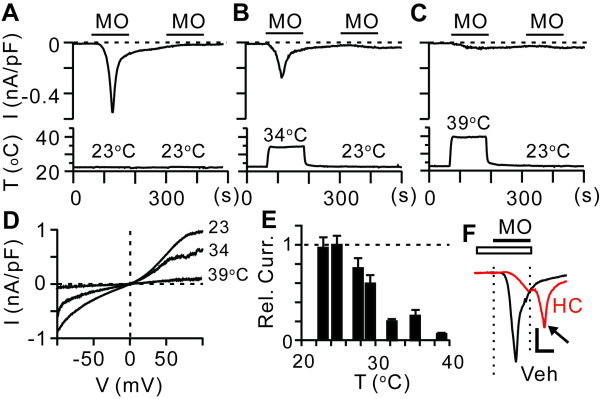
**Effects of warming on the activation of TRPA1 by mustard oil (MO) in HEK293 cells stably expressing rat TRPA1**. *A-C *(upper), current amplitudes measured at -80 mV by whole-cell voltage clamp recording in response to MO (100 *μ*M) at 23 (A), 34 (B) and 39°C (C); (bottom), changes in temperature throughout the recording. I, current density; T, temperature. *D*, representative current-voltage relationships of MO-evoked currents at the temperature indicated. V, membrane potential. *E*, temperature-response relationship of MO-evoked currents at various temperatures. Rel. Curr., relative current density normalized to the average current density at 23°C *n *= 10 in each bar. *F*, representative current traces evoked by 100 *μ*M MO with pretreatment and coapplication of vehicle (Veh, black) or HC030031 (10 *μ*M, HC, red) during the period indicated by a white bar. The arrow indicates an increase in current following washout of drugs. Dotted lines indicate the duration of MO application. Scale bar, 0.1 nA, 1 min.

Under our recording conditions, application of MO at 23°C induced robust activation followed by rapid attenuation of current amplitude even during the presence of MO. The acute desensitization accompanied strong tachyphylaxis such that repeated application of MO following initial activation did not evoke current any longer (Figure 1A). These activation and desensitization of TRPA1 are consistent with the properties reported by other groups [25-27]. To evaluate the effects of warm temperature on the MO-evoked activation of TRPA1, we increased bath temperature to 34°C or 39°C upon the application of MO. Surprisingly, the amplitudes of MO-evoked currents were markedly reduced at warm temperatures (Figure 1B-C). The current-voltage relationship demonstrated that warmth-induced suppression of MO-evoked currents was not dependent on membrane potential (Figure 1D). Suppression of MO-induced activation of TRPA1 was steeply regulated around the skin surface temperature, which is approximately 31°C (Figure 1E). A slight decrease or increase in temperature resulted in an increase or decrease, respectively, in the amplitude of MO-evoked currents. Warmth-induced suppression was almost complete at a temperature of 39°C, which is well below the level which evokes heat nociception in humans [28]. The application of MO at warm temperature not only reduced the initial activation but also induced strong tachyphylaxis. A second application of MO at 23°C following the first application of MO at 39°C did not evoke any currents either (Figure 1C). Heat-induced suppression was observed not only in a particular cell line stably transfected with rat TRPA1, but also occurred in HEK293 cells stably transfected by human TRPA1 or transiently transfected with human, rat and mouse TRPA1 (not shown). The warmth-induced suppression was as efficacious as HC030031, a specific inhibitor of TRPA1 [29], which induced 83 ± 6% inhibition at 10 *μ*M compared with vehicle (n = 7). In contrast to the effects of warming, inhibition by HC030031 was rapidly reversed upon washing out of drugs, resulting in an immediate increase in the current (at arrow in Figure 1F). These results suggest that innocuous warm temperatures can efficaciously suppress the agonist activation of TRPA1, and that warmth-induced suppression is not simply a result of the prevention or attenuation of activation, but may involve more complicated mechanisms, including desensitization of TRPA1.

**Figure 2:**
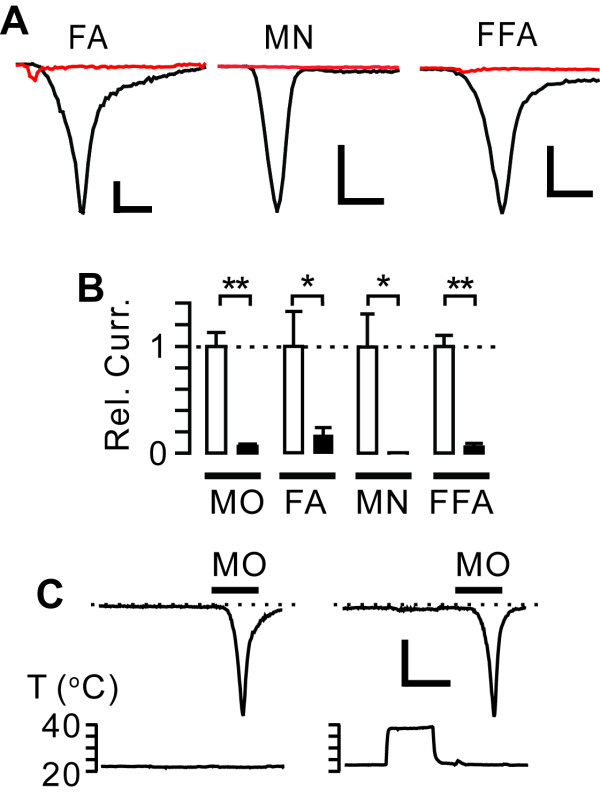
**Warming reduces agonist-induced activity of TRPA1**. *A*, example traces of currents evoked by formaldehyde (FA, 0.01%), menthol (MN, 250 *μ*M), flufenamic acid (FFA, 300 *μ*M) at 23°C (black) or 39°C (red). Superimposed traces were obtained from independent cells. Scale bar, 0.1 nA/pF, 30 s. *B*, normalized TRPA1 current densities at 23°C (white) and 39°C (black) induced by various agonists. *P < 0.05; **P < 0.001, *n *= 4-6 in each group. *C*, sample traces of currents evoked by 100 *μ*M MO (black bars) with or without pre-emptive heating as indicated. Scale bar, 0.2 nA/pF, 2 min. All data in panel A to C were obtained in rat TRPA1 except data for MN and FA in panel A and B that were obtained from human TRPA1.

Warmth-induced suppression was not unique to MO, but also occurred when another agonist, formaldehyde, activated TRPA1 (Figure 2A and 2B). The effects cannot be attributed to warmth-specific impairment of covalent modification by electrophilic agonists, because heat-induced suppression also occurred when non-electrophilic agonists such as flufenamic acid (FFA) [30] or menthol [9] were used (Figure 2A and 2B). Importantly, pre-emptive warming (39°C, 2 min) without co-application of MO neither evoked any current nor decreased the activation of TRPA1 when followed by application of MO (100 *μ*M) at 23°C (Figure 2C; -362 ± 50 pA/pF in control vs *-*337 ± 71 pA/pF in the heated group, *n *= 11, *P *> 0.7), suggesting that warmth does not alter the functionality of TRPA1 in the resting state.

### Warmth decreases apparent affinity and enhances desensitization of TRPA1

**Figure 3:**
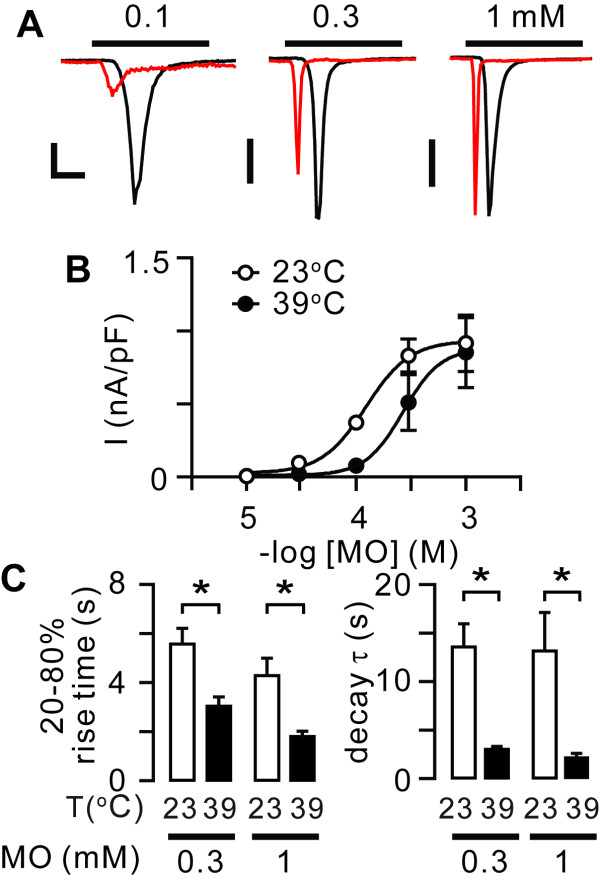
**Effects of warming on the apparent affinity of TRPA1 to MO and time course for activation and desensitization**. *A*, superimposed whole-cell current traces at -80 mV recorded from HEK293 cells stably expressing rat TRPA1 at 23°C (black) or 39°C (red) in response to 0.1, 0.3 and 1 mM MO. Superimposed traces were obtained from independent cells. Scale bars, 30 s, 0.1 (left), 0.3 (middle) and 0.5 nA/pF (right). *B*, concentration-dependent relationship of MO-evoked currents at 23°C (white) and 39°C (black). *C*, 20-80% rise time (left) and decay time constant (τ) (right) of currents evoked by 0.3 or 1 mM MO at 23°C (white) and 39°C (black). *P *< 0.05 in two-way ANOVA; *P < 0.05 in Bonferroni post-hoc test; *n *= 4-6.

Since cold induces or enhances TRPA1 gating [2,20,31], it is possible that a counter agonistic heat stimulus reduces agonist activation. When we compared the effects of warming following the application of various concentrations of MO, the suppressive effects became weaker as the concentration of MO increased (Figure 3A). When we quantified peak amplitudes, EC_50 _increased by ~2 fold from 122 *μ*M (95% CI: 69-217 *μ*M) at 23°C to 268 *μ*M (138-522 *μ*M) at 39°C. However, there was little effect on hill slope (2.1 at 23°C vs 2.5 at 39°C), top plateau (934 pA/pF at 23°C vs 883 pA/pF at 39°C) and bottom plateau (27 pA/pF at 23°C vs 12 pA/pF at 39°C). In this analysis, it was evident that warmth accelerated the time courses of current activation and desensitization as evidenced by the significant reduction of 20-80% in rise time and decay time constants (τ) at 39°C (Figure 3C). In particular, τ was greatly affected and reduced by 4-fold upon warming at 1 mM MO. Consequently, the mean area under the curve of MO-evoked currents decreased upon warming by approximately 3-fold at 1 mM (14.5 ± 0.2 at 23°C vs 5.1 ± 0.6 at 39°C, *n *= 6, *P *< 0.005). These results suggest that warmth reduced the apparent affinity of TRPA1 for MO and enhanced the rate of desensitization.

**Figure 4:**
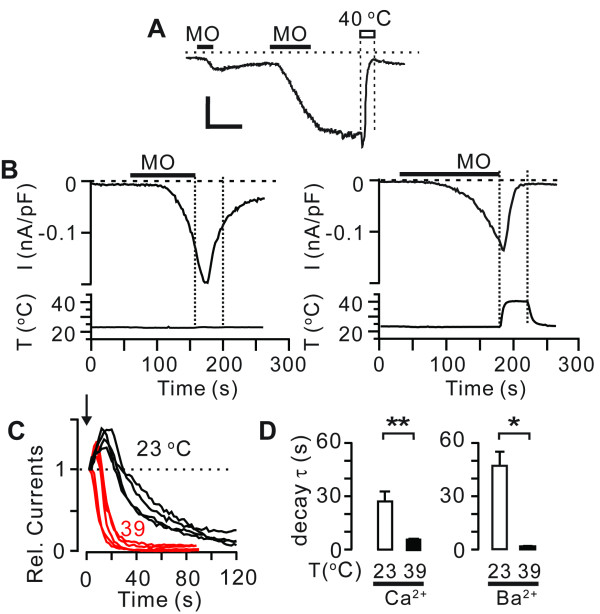
**Effects of warming on acute desensitization of TRPA1**. *A*, an example of the effects of heat following two consecutive application of MO (30 *μ*M) in a HEK293 cell transiently transfected by rat TRPA1. Scale bar, 3 min, 1 nA. *B*, representative current traces demonstrating the effects of temperatures on desensitization of rat TRPA1 following MO-evoked activation at 23°C. Currents were obtained from HEK293 cells stably expressing rat TRPA1. Washout of MO was performed at 23°C (left) or 39°C (right). The changes in temperature during recordings are shown in lower panels. Vertical dotted lines represent equivalent duration of time following washout of MO in different temperature as indicated. *C*, Examples of relative current traces recorded during the period between dotted lines in panel B obtained from multiple cells. The arrow indicates the time point at which the exposure to different temperatures was initiated. *D*, decay τ measured from the experiments in panel B in the presence of extracellular Ca^2+ ^or Ba^2+ ^at 23 (white) or 39°C (black). *, *P *< 0.05; **, *P *< 0.005 in Student's *t *test; *n *= 4-6.

Although warming affects the rate of MO-evoked activation of TRPA1, warmth-induced enhancement of desensitization may not entirely result from warmth-induced acceleration of the time course of activation. In HEK293 cells transiently transfected with rat TRPA1, MO-evoked activation was often not uniform but currents were frequently sustained even following washout as exemplified in Figure 4A. Such sustained currents were immediately suppressed by increasing temperature (Figure 4A). To assess the effects of warmth on the rate of desensitization independent of the rate of activation, we compared the rate of current decay when the cells were exposed to either 23 or 39°C following the activation by MO at 23°C. For better quantification, we used the HEK293 cells stably expressing rat TRPA1. When MO application was followed by warming to 39°C, the decay of the currents occurred immediately, with a significantly faster time course than that at 23°C (Figure 4B-D). Second application of MO at 23°C did not evoke any currents in both groups (not shown). Such warmth-induced decay was not dependent upon external Ca^2+ ^since warmth-induced suppression of TRPA1 occurred similarly in the presence of external Ba^2+ ^instead of external Ca^2+ ^(Figure 4D). Furthermore, even when the Ca^2+ ^level was strongly buffered by intracellular BAPTA, warmth -induced suppression of TRPA1 also occurred (not shown).

**Figure 5:**
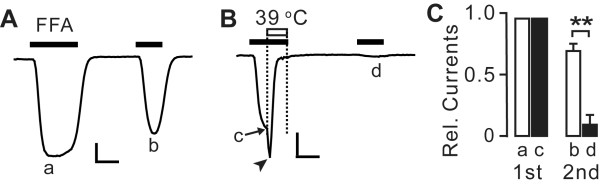
**Effects of warming on tachyphylaxis of TRPA1**. *A-B*, representative current traces evoked by two consecutive applications of FFA (300 *μ*M) in two independent HEK293 cells stably expressing human TRPA1. Initially FFA was applied at 23°C for 30 s. During the later half of the first application, FFA was applied at either 23°C (A) or 39°C (B). The second application was performed at 23°C in both groups. The letters a to d indicate time points for the quantification shown in panel C. The arrowhead indicates transient potentiation of TRPA1 currents by heat under this recording condition. Scale bar, 30 s, 4 nA (left), 2 nA (right). *C*, averaged relative current responses measured at points a to d in panel A and B. Current amplitudes were normalized to the respective peak amplitude during the initial half of the first application of FFA at 23°C. Black and white bars represent groups with or without heat co-application, respectively, during the first FFA application. **P < 0.001 in Student's *t *test; *n *= 5.

To further test the notion that warmth enhances desensitization of TRPA1, we tested whether heat facilitated tachyphylaxis. To attenuate tachyphylaxis of TRPA1 upon repeated agonist application, we used a divalent cation-free recording solution. To suppress endogenous background currents under these conditions, NMDG was used as a major charge carrier in the external solution. Like TRPV1 [32], TRPA1 shows permeation with NMDG [33]. Under these conditions, TRPA1 can be activated by repeated application of FFA with only a slight tachyphylaxis (Figure 5A and 5C). To test whether warmth enhances tachyphylaxis, we applied heat following the achievement of the apparent peak during the first application of FFA at 23°C (Figure 5B). Warming strongly suppressed the amplitude of FFA-induced currents by 95%. The second application of FFA at 23°C in this group evoked only small amplitude currents, which were significantly smaller than that in the non-heated group (Figure 5C). Of note, under this recording condition, warming during FFA application induced suppression preceded by transient potentiation of currents (arrowhead in Figure 5B), which was not obvious when Na^+ ^was used as a major charge carrier (Figure 4A and 4B). Overall, these results strongly support the idea that warmth-induced suppression of TRPA1 accompanies enhancement of desensitization.

We also examined whether warming suppressed the MO-induced activation of the mutant TRPA1 N855S that underlies familial episodic pain syndrome [6]. Like wild-type TRPA1, the amplitude of currents evoked by 100 *μ*M MO in HEK293 cells transiently transfected with TRPA1 N855S was significantly reduced by approximately 70% at 33°C compared with the amplitude at 23°C (-262 ± 61 pA/pF and -847 ± 142 pA/pF, respectively, *P *< 0.05).

**Figure 6:**
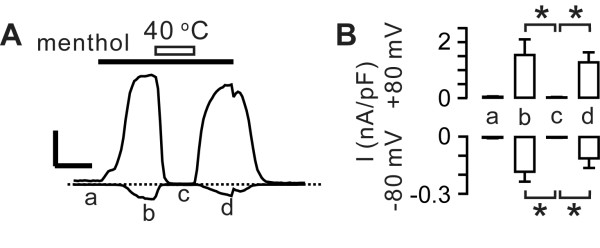
**Effects of warming on cold- and menthol-gated channel TRPM8**. *A*, representative current traces induced by menthol (300 *μ*M, black bar) from a HEK293 cell transiently transfected with rat TRPM8. Letters a to d represent the time points for quantification shown in panel B. Ca^2+ ^in the external solution was replaced with Ba^2+^. Scale bar, 0.5 nA/pF, 30 s; Vm = ± 80 mV. *B*, averaged current density at the indicated voltages. *P < 0.05 in Mann-Whitney rank-sum test; *n *= 4.

We tested whether warmth-induced suppression and desensitization also occurred in another cold-gated channel, TRPM8. In HEK 293 cells transiently transfected with TRPM8, menthol-evoked currents were almost completely suppressed when menthol was superimposed by a heat stimulus at 40°C. However, unlike TRPA1, the amplitudes of menthol-evoked currents were reversed approximately to the pre-heating level upon returning to room temperature (Figure 6). These results suggest that warmth may commonly suppress the agonist activation of TRPM8 and TRPA1, but only enhance desensitization of TRPA1 and not TRPM8.

### Innocuous warm temperature suppressed MO-induced ionic currents in sensory neurons

**Figure 7:**
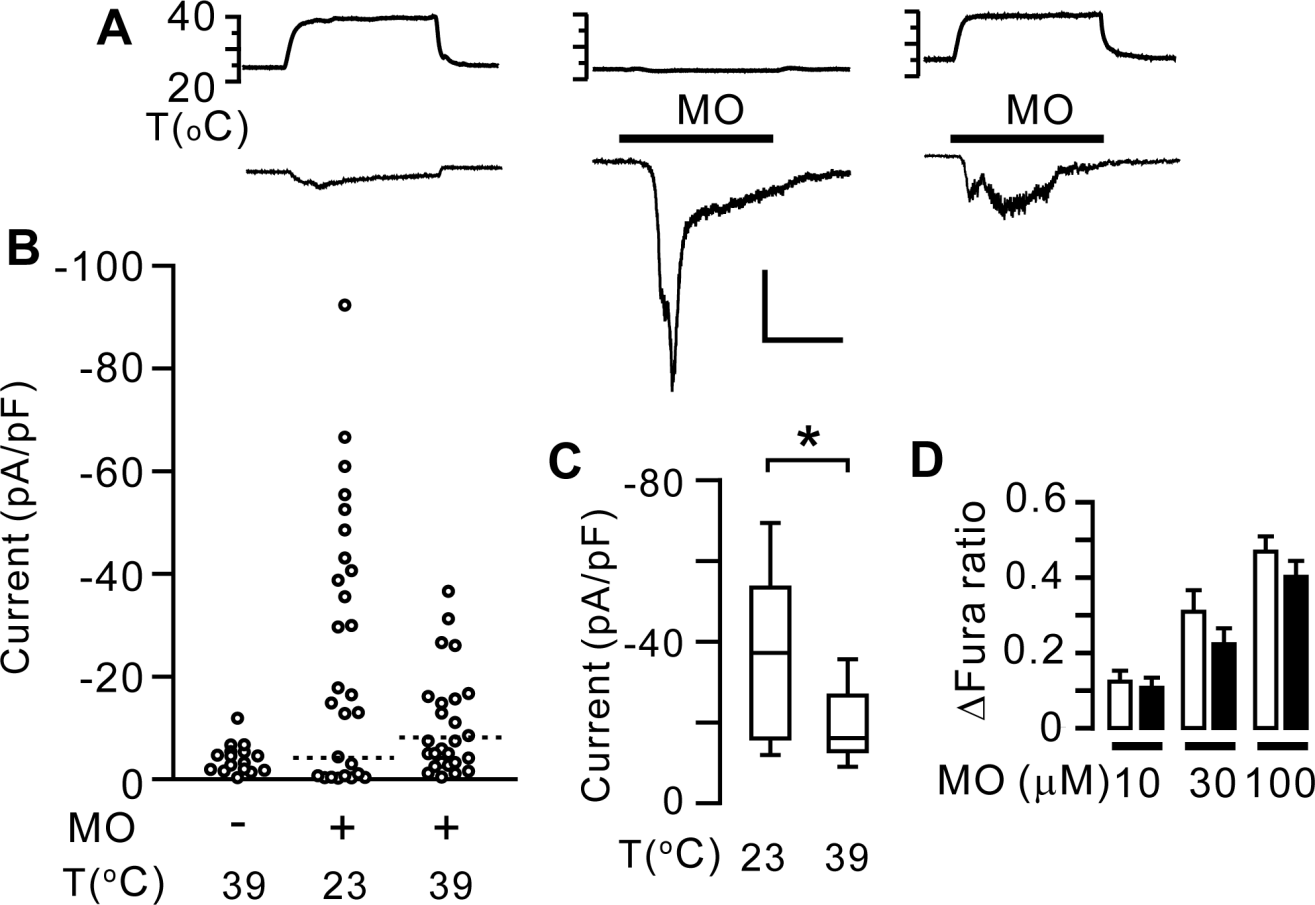
**Effects of warming on MO-induced ionic currents and intracellular Ca^2+ ^level in rat trigeminal ganglia (TG) neurons**. *A *(bottom), representative traces evoked by warming (left), MO (300 *μ*M) at 23°C (middle) or at 39°C (right) in cultured TG neurons; (upper), temperature during the equivalent periods. Vm = -70 mV. Scale bar, 0.5 nA, 1 min. *B*, data derived from experiments in panel A. Dotted lines represent the threshold determining responsiveness of each group. *C*, box-and-whisker plot comparing MO-evoked current densities at 23°C and 39°C. Only responders in panel B were quantified. Error bars, 5th to 95th percentile; *P < 0.05 in Mann-Whitney *U *test; *n *= 18 at 23°C and *n *= 11 at 39°C. *D*, changes in Fura ratio evoked by MO applied at 23°C with (black bars) or without (white bars) pre-emptive heat (39°C, 2 min). *P *> 0.14 in two-way ANOVA; 77-145 neurons/group.

Next, we determined whether warmth-induced suppression also occurred in native TRPA1 by recording MO-evoked currents in cultured rat trigeminal ganglia (TG) neurons. Warming to 39°C without MO only modestly changed the baseline current (4 ± 0.7 pA/pF, *n *= 16). MO (300 *μ*M) at 23°C evoked currents in 18 out of 23 neurons. When MO was applied at 39°C, however, only 11 out of 26 neurons responded (P < 0.05 in Fisher's exact test). The amplitude of the MO-evoked current was significantly attenuated in the 39°C group compared with the 23°C group (Figure 7A-C). As with recombinant TRPA1, however, pre-emptive warming did not affect the activation of TRPA1 by MO. Neuronal responses by the application of MO (10, 30, and 100 *μ*M) at 23°C was not significantly different between two groups of neurons with or without pre-exposure to warming at 39°C for 2 min (Figure 7D).

## Discussion

The major finding of this study is that agonist-induced activation of TRPA1 is strongly inhibited by innocuous warmth. Such inhibition is mediated by suppression, an attenuated activation, as well as desensitization, an inability of activation following initial activation. Our results suggest that heat reduces the functionality of TRPA1 only after activation by agonists. Pre-emptive heating without co-application of chemical agonists did not affect functionality of recombinant or native TRPA1, indicating that warmth by itself does not non-specifically impair the function of TRPA1. Heat-induced suppression of TRPA1 is apparently not the result of impaired covalent modification. Indeed, the rate constant representing the nucleophilic reaction between cysteine and hydrogen peroxide increases as temperature increases [34].

One plausible mechanism of heat-induced suppression of TRPA1 is that heat may act as a 'negative' modulator of TRPA1. In this model, agonistic temperatures positively modulate, whereas counter thermal stimuli may negatively modulate thermosensitive TRP channels. Indeed, agonist activation of heat-gated TRPV1 is strongly suppressed by cold, the counter agonistic temperature [35]. Although the time course of activation by MO was accelerated by heating, the EC_50 _increased at warm temperatures. In contrast, the heat-gated channel TRPV1 shows decreased EC_50 _upon heating [36]. Since TRPA1 can be gated and potentiated by cold [20], heat may act as a negative and positive modulator of TRPA1 and TRPV1, respectively. Recently, it was demonstrated that capsaicin- and heat-evoked activation may occur through distinct structural entities, and TRPV1, with simultaneous N628K/N652T/Y653T mutations, showed impaired activation by heat with intact activation by capsaicin [37]. Interestingly, this mutant TRPV1 also showed impairment in heat-induced potentiation and diminished cold-induced suppression of capsaicin-evoked currents [38], suggesting that the impairment of the heat activation pathway also attenuates cold-induced suppression. Therefore, it is also possible that the decreased EC_50 _of TRPA1 by warmth may be, at least in part, a result of negative modulation involving the cold activation mechanism of TRPA1.

Another interesting finding was that heat enhanced desensitization and tachyphylaxis of TRPA1 following agonist activation, but not that of TRPM8. The effects of heat on desensitization of TRPA1 cannot be fully explained by the accelerated activation time course. It is not clear how heat induces desensitization of MO-evoked currents, and the mechanisms need to be further investigated in the future. Importantly, heat-induced desensitization of TRPA1 is not the consequence of thermal activation of TRPA1. Although the structural basis of cold-induced activation/potentiation and heat-induced suppression/desensitization of TRPA1 is completely unknown, we speculate that thermal activation and desensitization may represent independent processes. Related to this notion is the finding that the TRPA1 orthologue from insects (drosophila and mosquito) and snake are activated by heat [39-41]. Drosophila TRPA1 is not only activated by heat but is also desensitized when it is expressed in HEK293 cells (M.K. Chung, unpublished observation). This is consistent with heat-induced activation and desensitization of another insect TRPA1 orthologue, a honeybee Hymenoptera-specific TRPA [42]. The heat-desensitizing mechanism of drosophila and mammalian TRPA1 may be evolutionarily conserved, while thermal activating mechanisms are not. Further structure-function studies are required to investigate the distinct region involved in thermal activation and heat desensitization of these channels. Alternatively, it is possible that the agonist-induced desensitization process is highly sensitive to elevated temperature and heat enhances the rate of desensitization induced by agonist. Investigation of the detailed mechanisms of TRPA1 desensitization and their temperature sensitivity may provide further mechanistic insights.

Since desensitization of TRPA1 is related with the internalization of channel proteins [43], we cannot exclude the possibility that internalization of TRPA1 by warming could contribute the warmth-induced inhibition of TRPA1 activity. However, we think that the warmth-induced inhibition of TRPA1 cannot be mainly due to such mechanism based upon two reasons: 1) Current amplitudes evoked by 1 mM MO were comparable between 23 and 39°C, suggesting the equivalent existence of functional TRPA1 at the surface of plasma membrane, and 2) the decay time constant of inhibition was as small as < 5 sec, which may be too fast for the internalization of the entire channels in the plasma membrane. Further biochemical and cell biological approaches are required to clarify the mechanisms of warmth-induced effects on TRPA1.

Mild heating not only suppressed recombinant TRPA1, but also attenuated MO-evoked ionic currents in TG neurons, suggesting that warmth suppresses the activation of endogenous TRPA1. We cannot exclude a possibility that MO-induced responses partly involve other molecules, e.g., TRPV1 [44]. Indeed, we also observed a weak activation of TRPV1 by a high concentration of MO and superimposition of MO on warmth potentiated currents (M.K. Chung, unpublished observation). Therefore, MO-evoked currents at warm temperature in sensory neurons could be partly the result of activation of TRPV1. However, such agonistic effects of MO on TRPV1 cannot explain heat-induced suppression of MO-evoked currents in TG, which strongly suggest substantial suppression of agonist-induced activation of TRPA1 in nociceptors.

Persistent activation of TRPA1 may contribute to the maintenance of persistent pain at the site of injury or inflammation [14,16]. We demonstrated that innocuous heating suppressed MO-induced nociceptor activation. Tissue temperature has been found to increase by approximately 3 - 4°C at the site of acute inflammation in experimental animals [22,23], which may substantially attenuate activation of TRPA1 by putative endogenous agonists. Therefore, local hyperthermia during acute inflammation may be an endogenous anti-nociceptive mechanism preventing excessive pain at the site of injury. Also heating of local tissues or the whole body is a remedy that has been widely used for attenuating chronic pain conditions, such as low back pain and osteoarthritis [45-47]. Our results suggest that heat-induced suppression of TRPA1 may provide, in part, a peripheral molecular mechanism of heat therapy. It is worthwhile to note that warmth desensitizes TRPA1 only when the channel is activated, but not in the resting state (Figures 2C and 7D). Thus innocuous heating may preferentially desensitize TRPA1 in an active but not a resting state, thus preserving the role of TRPA1 as a sensor of tissue damage.

In conclusion, we demonstrated that TRPA1 is modestly activated and potentiated by cold, but is strongly suppressed by innocuous heat. Warmth-induced suppression is a result, not only of reduced activation, but also of enhanced desensitization. These results may provide a novel insight for thermosensitivity of TRPA1 and a mechanistic rationale for the application of mild heating to attenuate nociception.

## Methods

### Cell culture and transfection of cDNA

Rat TRPA1, human TRPA1, rat TRPM8 cDNAs were generously gifted by Dr. David Julius (University of California, San Francisco, CA). Mouse TRPA1 cDNA was kindly gifted by Dr. Ardem Patapoutian (Scripps Research Institute, San Diego, CA). To generate the human TRPA1 N855S mutant, site directed mutagenesis was performed by overlap extension polymerase chain reaction methods using a pair of specific primers.

HEK293 cells were maintained as previously described [38]. Cells were maintained in Dulbecco's Modified Eagle Medium (DMEM) containing 10% fetal bovine serum and penicillin/streptomycin/glutamine (PSG) in 5% CO_2 _at 37°C. Lipofectamine-2000 (Invitrogen) was used for the transient transfection of cDNA. To identify transfected cells, cDNA encoding mCherry was co-transfected. HEK293 cells stably transfected with rat or human TRPA1 were generated by selection of transfected cells with G418. Resistant clones were screened for functional expression of TRPA1 by electrophysiology and constitutive expression of mRNA of TRPA1. Transfected HEK293 cells were plated onto polyornithine-coated round coverslips (8-mm in diameter) and used for experiments after 16 to 24 hours.

### Culture of TG neurons

Rat TG neurons were dissected as described previously [38]. All procedures were conducted using twelve male Sprague-Dawley rats (body weight 200-300 g, Harlan) in accordance with the NIH *Guide for the Care and Use of Laboratory Animals *and under a University of Maryland-approved Institutional Animal Care and Use Committee protocol. Rats were euthanized using a lethal dose of sodium pentobarbital. The ganglia were dissected out and minced in cold DMEM/F12 containing 10% horse serum and PSG. TG were incubated in 1 mg/ml collagenase (type XI, Sigma) for 30 min at 37°C and triturated with flame-polished Pasteur pipettes. The ganglia were incubated in phosphate-buffered saline (PBS) containing 0.05% trypsin and 0.1% EDTA for 2 min at 37°C. After washing with serum-containing culture medium, dissociated cells were added to a 25% Percoll gradient, centrifuged for 12 min at 900 g, and plated on to polyornithine- and laminin-coated glass coverslips. The neurons were cultured in DMEM containing 10% horse serum, 1% PSG, and 100 ng/ml nerve growth factor, in 5% CO_2 _at 37°C. The neurons were assayed 16-48 h later.

### Electrophysiology

Following previously described methods [32,38], conventional whole-cell patch clamp recordings were performed using the Axopatch 200B with Digidata 1440 interface (Molecular Devices). Unless otherwise indicated, the bath solution contained 140 NaCl, 5 KCl, 2 CaCl_2_, 1 MgCl_2_, 10 glucose, 10 HEPES (in mM, pH 7.4 adjusted with NaOH, 300-310 mOsm). The pipette solution contained 140 KCl, 5 NaCl, 10 EGTA, 1 CaCl_2_, 1 MgCl_2_, 2.5 Mg-ATP (in mM, pH 7.3 adjusted with KOH, 290-300 mOsm). As indicated in the Results section, external Ca^2+ ^was replaced with Ba^2+ ^in some experiments. To reduce tachyphylaxis of TRPA1 activation in Figure 5, we used divalent-free internal and external solutions containing 150 X-OH, 10 HEPES, 10 EDTA (X = Na in the internal and N-methyl-d-glucamine (NMDG) in the external solution; pH 7.4 adjusted with HCl, 290-300 mOsm). Osmolarity of every solution was measured using a vapour pressure osmometer (Wescor) and adjusted using mannitol as necessary. Borosilicate glass electrodes with tip resistances of 2-4 MΩ were fabricated using a pipette puller (Sutter). Series resistance was compensated > 75%. A 3 M KCl agar salt bridge was used throughout the experiments. To evaluate current amplitudes and current-voltage relationships at the same time in HEK293 cells, we applied repetitive 200 ms voltage ramp pulses (-100 to +100 mV, 1 mV/ms at 0.5 Hz) from a holding potential of 0 mV. Voltage protocols were delivered and currents were acquired using Clampex (Molecular devices).

In sensory neurons, membrane potential was continuously clamped at -70 mV. Following the application of MO, neurons showing currents greater than -4 pA/pF, which was twice the mean rms noise, were regarded as responders. In groups exposed to 39°C heat, the threshold was determined as -8 pA/pF, which was twice the mean heat-induced change.

Bath temperature was controlled using an in-line heater (Warner instruments) connected to a valve-controlled perfusion system driven by gravity. Actual bath temperature was monitored continuously with a thermocouple (IT-18, Physitemp) placed within 4 mm of the patch-clamped cell and recorded in Clampex throughout the experiment. The bath (< 200 *μ*l) was continuously perfused at a rate of ~3 ml/min. Temperature values referred to in the results section represent the mean of the actual temperatures measured for each group. In this study, we used the term 'warmth' to indicate an innocuous range of heat above that of the skin surface temperature, i.e., approximately 32°C to 40°C.

### Ratiometric Ca^2+ ^imaging

Ratiometric Ca^2+ ^imaging experiments and analysis were performed as described previously [48] with slight modifications. Sensory neurons were loaded with 1 *μ*M fura-2 acetoxymethyl ester (Anaspec, Inc) with 0.01% pluronic acid (Anaspec, Inc) for 40 min at 37°C in Ca^2+ ^imaging buffer containing 130 NaCl, 3 KCl, 2.5 CaCl_2_, 0.6 MgCl_2_, 10 HEPES, 10 sucrose, 1.2 NaHCO_3 _(in mM, pH 7.45, 310 mOsm after adjustment with mannitol). Measurement of fluorescence was performed using an inverted fluorescence microscope (Nikon) equipped with a filter changer (Sutter Instruments) and a CCD camera (Nikon). Paired images (340 nm and 380 nm excitation, 510 nm emission) were collected every 2 s and the fura ratios (emission at 340 nm excitation/emission at 380 nm excitation) were calculated. Data acquisition and analysis were performed using NIS Elements (Nikon).

### Reagents

MO (allylisothiocyanate, Sigma) was diluted in DMSO for cellular electrophysiological experiments. Stocks of HC030031, menthol and flufenamic acid (Sigma) were dissolved in DMSO and diluted in the external solution. Formaldehyde (37%, Fisher) was diluted directly in the external solution.

### Data analysis and statistics

To minimize variability due to the differences in individual cell size, we determined current densities (current amplitude/membrane capacitance). Statistical analysis was performed using Prism or SigmaStat. Curve fitting of current traces was performed using pClamp (Molecular Devices). Concentration-dependent relationships were fitted by a logistics function: *Y *= *Min *+ (*Max*-*Min*)/(1 + 10^(Log*EC*_50 _- *X*) × *H*) where *Y *= current density, *Min *and *Max *= Minimum and maximum plateaus, *EC*_50 _= half-maximal concentration, *X *= log of concentration, *H *= Hill slope. The curves were fitted without constraining the four parameters. Data in all figures represent mean ± s.e.m. Unless otherwise indicated, the Student's *t *test was used to compare two groups and *P *< 0.05 was considered to be statistically significant.

## Abbreviations

DMEM: Dulbecco's Modified Eagle Medium; FA: Formaldehyde; FFA: Flufenamic acid; 4-HNE: 4-hydroxynonenal; HEK: Human embryonic kidney; MO: Mustard oil; NMDG: N-methyl-d-glucamine; PSG: Penicillin/Streptomycin/Glutamine; TRPA1: Transient receptor potential A1; TRPV1: Transient receptor potential v1; TG: Trigeminal ganglia.

## Competing interests

The authors declare that they have no competing interests.

## Authors' contributions

MKC, SW, JL performed the experiments. All authors contributed to the design of the experiments and analysis of the data, and approved the final version of the manuscript.
